# Rotating Bending Fatigue Analysis of Printed Specimens from Assorted Polymer Materials

**DOI:** 10.3390/polym13071020

**Published:** 2021-03-25

**Authors:** Marino Brčić, Sanjin Kršćanski, Josip Brnić

**Affiliations:** Department of Engineering Mechanics, Faculty of Engineering, University of Rijeka, Vukovarska 58, HR-51000 Rijeka, Croatia; sanjink@riteh.hr (S.K.); brnic@riteh.hr (J.B.)

**Keywords:** rotating fatigue, fused filament fabrication (FFF), PLA, ABS, ASA

## Abstract

Fused filament fabrication (FFF), as a form of additive manufacturing (AM), in recent years, has become a popular method to manufacture prototypes, as well as functional parts. FFF is an extrusion process, commonly known as 3D printing, where the object is built by depositing melted material layer by layer. The most common materials, i.e., the materials that are most widely used, are polylactic acid (PLA), acrylonitrile butadiene styrene (ABS) and acrylonitrile styrene acrylate (ASA). Although there are lot of research papers that cover the subject of the determination of mechanical properties and characteristics, theoretically and experimentally, as well as the fatigue characteristics of aforementioned materials, there is a lack of research and scientific papers dealing with the problematics of S–N curves based on the rotating bending fatigue analysis of those materials. Consequently, this paper covers the topic of rotating bending fatigue data for 3D printed specimens of given materials, under different loading values.

## 1. Introduction

Additive manufacturing (AM) technologies are, according to American Society for Testing and Materials (ASTM) Standard F2792-12a [1], defined as a process of joining materials to make objects from a 3D model, as opposed to subtractive manufacturing methodologies. The layer-by-layer technique is a feature that gives the AM methods an advantage over traditional manufacturing processes, especially for rapid prototyping (RP), where the primary intention is to fabricate models for visualization, design verification and model improvement [2]. Nevertheless, recently AM techniques emerged as a processes where the objective is the production of finished components and products [3]. 

The most popular AM technique is fused filament fabrication (FFF), also known under trademarked name fused deposition modelling (FDM), classified under material extrusion processes, where material is dispensed through a nozzle [1]. The popularity of the FFF process has grown with the rapid development of commercially available 3D printers and computer aided design (CAD) software. 3D printing, or FFF technology, has another big advantage over other technologies, which is fast, effective and adaptive tool development, in small series production. As mentioned previously, it has proven essential in emergencies, such as the global pandemic caused by COVID-19. For example, the 3D printing and other AM technologies were globally used for the production of personal protective equipment (PPE) for healthcare and medical personnel [4]. The authors of the paper at hand also participated in such commendable action.

The FFF process is briefly given, as follows. The product, i.e., the CAD model, is prepared and exported in stereolithography (STL) file format and uploaded into slicer software, which thus prepares the G-code file format, recognizable by 3D printer. The polymer material filament is then heated to a specified temperature and pushed through, or better said, extruded through the nozzle onto the heated build platform, thus forming one layer of future product [5]. By researching available scientific works, papers and articles, three materials stand out as the most popular materials used by FFF techniques. These are: polylactic acid or polylactide (PLA); acrylonitrile butadiene styrene (ABS); and acrylonitrile styrene acrylate (ASA). PLA belongs to natural thermoplastic biodegradable polymer, derived from renewable raw materials, e.g., sugarcane, starch and corn [6]. More specifically, PLA belongs to an enantiomeric polyester group of polymers, which includes L-lactic acid and D-lactic acid [5] and is a biodegradable polymer, approved for various biomedical applications [7]. PLA’s biodegradability can be divided into microbial and enzymatic degradation [8]. It is also interesting as a material for medical application in bone reconstruction [9]. Another PLA advantage is its low melting temperature, which requires less energy to print, but is also very brittle [10]. ABS is thermoplastic copolymer, widely used in the industry (automotive, electronic, etc.), especially for the injection of molded components, but can be more difficult to print due to layer bonding and warping issues [11]. Although it has better mechanical properties and chemical resistance than PLA, its application is limited by its easy flammability, which is, in turn, sustained by the incorporation of bromine flame retardants [12]. However, this leads to the release of toxic substances and corrosive smoke and to increasing health and environment issues [11]. Comparing ABS to ASA material, ASA is similar to ABS in terms of mechanical properties and printing parameters, but since ASA uses saturated weather-resistant acrylate rubber in place of unsaturated butadiene rubber, ASA is of the weather-resistant polymer class [13]. This fact makes ASA a UV-stable thermoplastic, with long-term heat and weather resistance, thus suitable for housings and covers for various outdoor applications [14]. Like ABS, ASA also has an excellent surface finish, high-impact resistance and stiffness. 

Dickson et al. [15] also stated that the three mentioned materials are the most popular FFF materials, but also emphasize that the polymer product can lack the strength crucial for fully functioning engineering parts. Thus, to overcome this, reinforcing materials are used, i.e., reinforcing materials such as fibers, which are added to the polymers and polymer matrix during FFF process. Several authors report similar principles, in which adding the reinforcing material actually produces a composite structure, which in turn exhibits improved mechanical properties. In [16], Der Klift et al. present the research on carbon fiber-reinforced thermos-plastic (CFRTP) composites, used for structural applications in aerospace engineering and automotive industry. The work of a group of authors led by Khatri et al. [17] presented the ABS matrix barium titanate (ABS-BT) composite, compatible with commercially available 3D printers. The mentioned composite specimens showed an increase in elastic modulus value by up to 69% compared to pure polymer specimens. Another interesting scientific work is presented in [18], where a review of the proposed modified polymer composites, associated with rapid prototyping and 3D printing, is given. Not only synthetic fibers are used as reinforcement in FFF composite filaments, but also natural fiber-reinforced polymer composites, as stated in [19]. Stoof et al. [19] reported the results of a tensile test on PLA polymer composites reinforced with hemp and harakeke fibers, in varying percentages, and an increase in the Young’s modulus value in comparison to plain PLA.

During the AM process, as in the FFF process, material undergoes a fundamental phase transformation [20], changing geometry and mechanical properties. Parts produced using FFF exhibit significant material anisotropy and heterogeneity, a fact that cannot be neglected. In addition, there are many parameters to control during the FFF process, which make the analysis very complex. It is, thus, very important to understand the mechanical properties and characteristics of FFF products and specimens, since those characteristics are crucial for final product customization and usage. Therefore, a large number of research papers are dedicated to this matter. The vast majority of studies are experimentally based, using commercially available 3D printers, and focused on the tensile strength and elastic properties of PLA [11,21,22] ABS [5,10,11,23,24,25] and ASA [11]. Aforementioned research papers are based upon different specimen patterns, raster angles and infill percentage. Cuan-Urquizo et al. [26] gave a review of several experimental characterizations of FFF structure approaches, but also gave a review of predictive analytical models. They concluded that almost no approach can be used individually and that the experiments can hide the individual influence of manufacture or structural parameter and the analytical approaches rely on assumptions. Thus, the experimental analysis, such as fatigue analysis, can result in a quicker estimation of mechanical performance. Ezeh and Susmel in [27] emphasized that giving quantitative recommendations of PLA fatigue will help engineers perform the fatigue assessment in practical situations. In that sense, authors presented the fatigue behavior analysis of AM PLA specimens, with the attention to the effect of raster orientation and infill level. This was similar to what was presented in [28] for the FFF-prepared ABS specimens, where the tension and tensile fatigue test results were given for different raster orientations. There is still a significant amount of quality research papers on the fatigue of FFF polymer specimens, but there is a lack of references on rotating fatigue or rotating the bending fatigue of 3D printed polymer specimens. Domingo-Espin et al. [29] give a rotating flexural fatigue test results on FFF ABS specimens, with emphasis on parameters such as layer height, printing speed, infill density and nozzle diameter. As a result, S–N curves are given, associated with the best 3D printing parameters. This is similar to what was presented by the same group of authors, in the paper [30], but for PLA specimens. Again, S–N curves and adjusted Wöhler model are given.

In accordance with all the aforementioned, and taking into account all the gathered facts, the paper at hand presents the rotating bending fatigue results, as well as the tensile test results, for 3D printed specimens made from the three most common FFF polymer materials, namely PLA, ABS and ASA. The paper emphasis is on rotating fatigue, and thus the results are given for different rotating speeds and dynamic bending loads in the form of simplified S–N fatigue diagrams.

## 2. Materials and Methods

The previous chapter provides a brief overview of part of the research in the field of AM and FFF processes, i.e., 3D printing. It was emphasized that the three polymers occupied a central place in most research works, both experimental and analytical. It is further stated that the presented paper will concentrate on the rotating, bending or flexural fatigue analysis of the 3D printed specimens of most popular polymers:

- PLA—manufacturer Shanghai Fusion Tech Co., Ltd. [31];
- ABS—manufacturer Shanghai Fusion Tech Co., Ltd. [32];
- ASA+—manufacturer PrimaCreator, Malmö, Sweden [33].

Some of physical properties of the used materials, provided by the manufacturer, are given in Table 1. In order to obtain necessary mechanical data of the polymer specimens needed for rotating fatigue, tensile tests were performed at room temperature for the mentioned materials. Specimens for tensile and fatigue tests were prepared using Raise3D printer (Raise3D, Irvine, CA, USA), model Pro 2 Plus. The nozzle of the 3D printer is set up for filaments of 1.75 mm diameter. Thus, the material properties given in Table 1 relate to filaments of this diameter.

### 2.1. Tensile Tests

**Figure 1:**
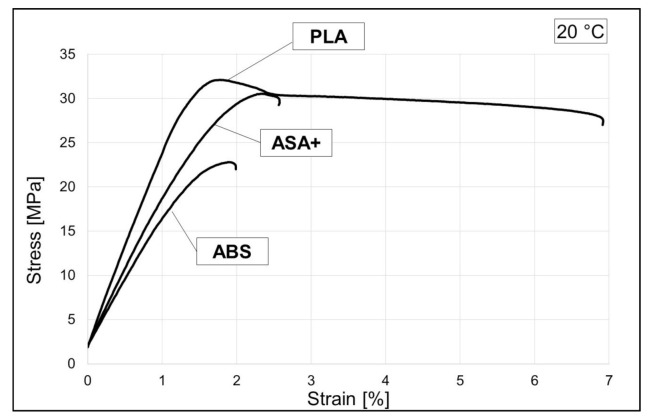
Engineering stress–strain diagrams at room temperature for polylactide (PLA), acrylonitrile butadiene styrene (ABS) and acrylonitrile styrene acrylate (ASA+) 3D printed specimens.

Tensile tests were conducted on a Zwick/Roell Z400 material testing machine (ZwickRoell, Ulm, Germany), at room temperature. Due to the available test equipment, round tensile specimens were prepared, with 8 mm diameter and 60 mm specimen measuring length. Printing parameters were set as follows. The infill density was set to 100% with a shell thickness of 2.0. Layer height was set at 0.2 mm, with grid infill pattern 0°–45°–135°. Print temperature was set at 205 °C for PLA and 250 °C for ABS and ASA+. Heated bed temperature also varies depending on the material, i.e., 60 °C for PLA specimens and 100 °C for ABS/ASA+ specimens. Raft and supports are added to every print specimen model, to prevent shrinkage and temperature induced bending. Regarding printing speed, the first layer printing speed was set at 15 mm/s, while the default printing speed was 50 mm/s. The tensile test results, in the form of stress–strain diagram, are given in Figure 1 and tabular, in Table 2, Table 3, Table 4, where the obtained results are compared with manufacturer data and other research results.

### 2.2. Rotating Fatigue Tests

**Figure 2:**
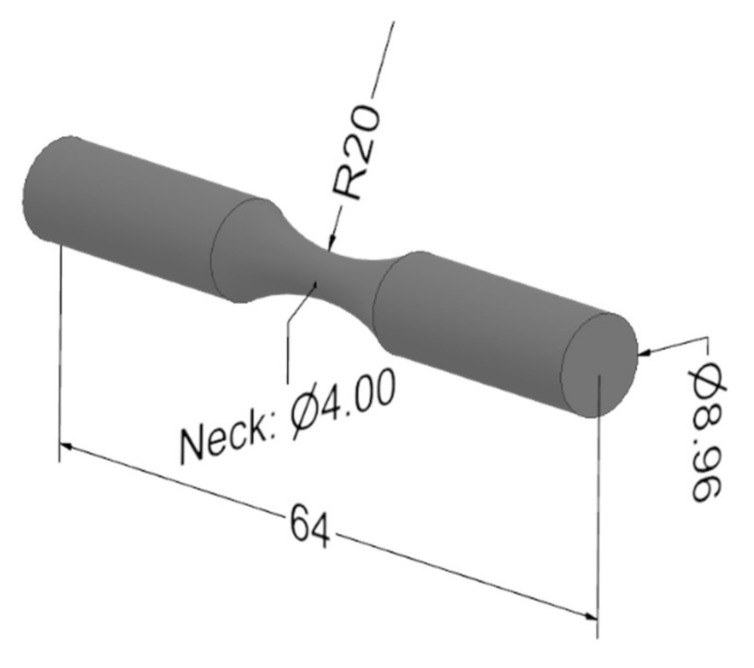
Rotating bending fatigue specimen, with the dimensions (mm).

The specimens for the rotating bending fatigue test were prepared in the same manner as for the tensile test, using the same printing equipment and setup. Since there is no specific standard, by authors’ knowledge, for laminated polymer and plastic fatigue test, the test specimens were prepared as depicted in Figure 2., i.e., as defined by the testing equipment manufacturer. The surface roughness was also an important parameter in fatigue life analysis. Scratched surfaces cause stress concertation, thus smooth surfaces provide a better stress distribution and reduce fatigue fracture. The surface roughness of FFF polymer specimens depends on two parameters, namely the layer height and nozzle diameter, and it can be determined either experimentally or calculated using the proposed term in [35] *R_a_* = 112.5 × *t*, where *R_a_* is the surface roughness in µm and *t* the layer height in mm. With the nozzle diameter of the 3D printer used for preparing samples equal to 0.2 mm with a layer height of 0.2 mm, the surface roughness of the used specimens was *R_a_* = 22.5 µm, which is in accordance with the surface roughness that can be found in the literature for similar FFF parameters and polymers [36].

**Figure 3:**
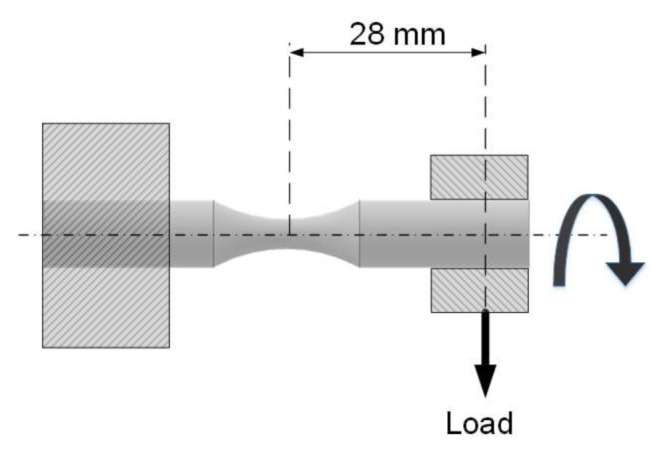
Rotating bending fatigue machine setup.

The rotating bending fatigue machine clamps the specimen as a cantilever, as shown in Figure 3. The test machine is TQ SM1090 (TecQuipment Ltd., Nottingham, UK) with versatile data acquisition system, which uses an adjustable dead weight for applying vertical, downward load on the specimen, using a self-aligning bearing inside a gimbal. The dead weight can be adjusted on the toothed leverage, which makes sure that the tests, i.e., the load on the specimen, are repeatable. The load on its free end creates tension on the upper half of the specimen, and likewise compression on the lower half. The test machine repeatedly stresses a test specimen for a known number of cycles, with alternate compressive and tensile stress on any given part along the unsupported length of the test specimen. The point inside the cross section of the specimen, except the point on the neutral axis, moves from zero stress to the maximum tensile stress and back through the zero stress and maximum compressive stress. This span represents one cycle with two reversals, where the reversal represents the path from the maximal positive to the maximal negative stress and vice versa. Thus, the applied stress on specimen vs. cycle is described with sinusoidal function.

The stress at the neck of the specimen can easily be calculated using the standard bending Equation (1), where *F* is the load on the free end of the specimen, and *l* is the distance from the neck to the load and *d* is the diameter at the specimen neck:

(1) $$\sigma =\frac{32\cdot F\cdot l}{d^{3}\cdot \pi }$$

## 3. Fatigue Test Results and Discussion

As mentioned in the first section of the paper, there is a shortage of research papers on a rotating bending fatigue of polymer materials and FFF specimens. Domingo-Espin et al. in [29] and Gomez-Gras et al. in [30] presented the experimental and numerical analysis results on the rotating fatigue of FFF specimens from PLA and ABS polymers. In both cases, the authors concluded that infill density shows the highest influence on fatigue performance, i.e., the fatigue life increases with the infill density value. However, since they observed only specimens with a maximum of 75% infill density, it is therefore interesting to study the fatigue life of specimens with 100% infill density. Hence, the paper at hand explores different stress levels of the rotating bending fatigue life of PLA, ABS and ASA specimens, with 100% infill density and a rectilinear (grid) pattern. In addition, different rotational speeds were applied for every specimen material, i.e., the 10 Hz, 20 Hz and 30 Hz cycle rate.

Using the obtained test results, the S–N curve model was prepared, for every material and cycle rate. The S–N curve model was based on the typical S–N curve (Equation (2)), based on the early Basquin model (Equation (3)) of S–N curves [37]:

(2) $$\sigma_{a}=\sigma_{f}^{\prime }{\left(2N_{f}\right)}^{b}$$

(3) $$\sigma_{m}=\alpha {\left(N_{f}\right)}^{\beta },$$

where *σ_a_* represents the constant stress amplitude in the equation where the fatigue life is given in the form of two reversals, 2*N_f_*, while *σ_m_* represents the peak stress in formulation with the given fatigue life, *N_f_*. $\sigma_{f}^{\prime }$ and *α* are the fatigue strength coefficients, while *b* and *β* represent the fatigue strength exponent. These last mentioned coefficients belong to the group of model-fitting parameters. The expression given in (3) is widely used in the fatigue analysis based on the stress approach. In order to obtain the mentioned typical S–N curve equation, the least-squares regression model (4) serves as a starting point:

(4) y=A+Bx

where *A* and *B* are obtained through values from test specimens:

(5) $$B=\frac{n\sum_{i=1}^{n}y_{i}x_{i}-\sum_{i=1}^{n}x_{i}\sum_{i=1}^{n}y_{i}}{n\sum_{i=1}^{n}x_{i}^{2}-{\left(\sum_{i=1}^{n}x_{i}\right)}^{2}}$$

(6) $$A=y_{avg}-Bx_{avg}$$

In the above equations, *x_i_* and *y_i_* are the measured values of stress and the number of cycles for the *i*th specimen, respectively, from altogether an *n* number of specimens, and *x_avg_*, *y_avg_* are the average values of the *x* and *y* series, given by $\sum x_{i}/n$ and $\sum y_{i}/n$, respectively. Equation (3) can be written in logarithmic form, taking logarithms with base 10 of both sides of the equation and rearranging it:

(7) $$\log\left(N_{f}\right)=-\frac{1}{\beta }\log\alpha +\frac{1}{\beta }\log\sigma_{m}$$

By comparing Equation (7) with Equation (4), the dependent (8) and independent (9) variables can be determined, as follows:

(8) $$y=\log\left(N_{f}\right)$$

(9) $$x=\log\sigma_{m}.$$

Similarly, the coefficients *A* and *B* can be expressed as

(10) $$A=-\frac{1}{\beta }\log\alpha$$

(11) $$B=\frac{1}{\beta }.$$

**Figure 4:**
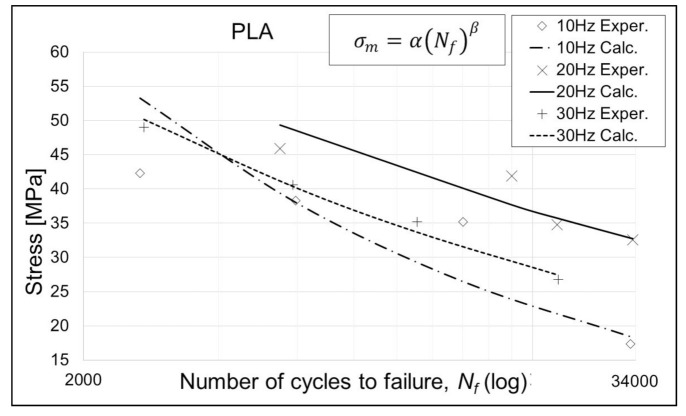
S–N curves for PLA specimens. Exper.—Experimental; Calc.—Calculated.

**Figure 5:**
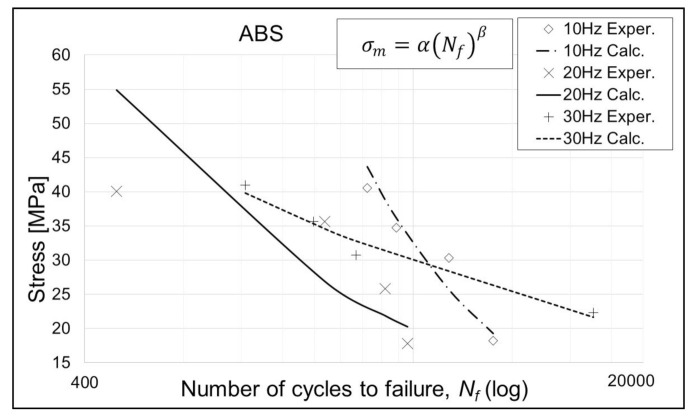
S–N curves for ABS specimens. Exper.—Experimental; Calc.—Calculated.

**Figure 6:**
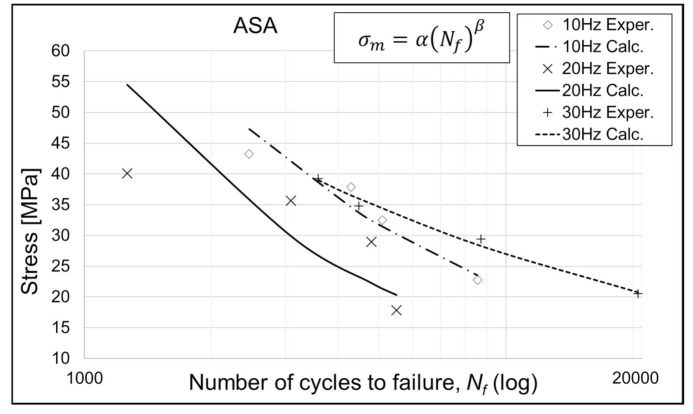
S–N curves for ASA+ specimens. Exper.—Experimental; Calc.—Calculated.

Coefficients *α* and *β* for the performed fatigue test, relevant to the S–N curve model presented by Equation 3, are given in Table 5, Table 6 and Table 7, while the curves modeled with the same equation are given in Figure 4, Figure 5 and Figure 6. It is evident, from the results and diagrams, that the proposed model is valid for low cycle fatigue, i.e., lower than 10^5^ cycles. It is also worth noting, and it can be clearly seen on diagrams, that specimens with the lowest applied stress value reached the failure.

## 4. Conclusions

This paper gives a brief overview of FFF-produced specimen tensile and fatigue experimental research, with an emphasis on rotating bending fatigue tests. As stated and shown, there is a lack of experimental and analytical research on rotating bending (flexural) fatigue on 3D printed polymer material specimens, so the intention of the paper at hand was to address this issue with the introduction of rotating bending fatigue analysis of FFF specimens of PLA, ABS and ASA+ materials. The tests were performed on 100% infill density polymer specimens, with a rectilinear pattern, in contrast to the referenced 75% infill, which can be found in the literature. Additionally, different rotational speeds, i.e., cycle rates, were considered. The obtained results were given in the form of S–N curves and a simplified analytical model.

It can be noticed from Figure 5 and Figure 6 that the ABS and ASA+ specimens exhibit very sensitive behavior at the 20 Hz cycle rate. PLA specimens do not exhibit such behavior, and since all specimens were prepared in the same manner, using same equipment and environmental conditions, this phenomenon demands additional research. Proposed research would include the eigenvalue analysis of a specimen model and additional fatigue analysis with a larger number of points, in the same stress level range. This can be achieved by modifying the specimen geometry, i.e., the specimen neck diameter. In that way, the range of bending stress can be distributed on several dead weight (load) positions on the machine leverage, and thus, larger numbers of points of S–N curves can be obtained.

As is emphasized, further research into 3D printed specimens, especially 3D printed polymer specimens, is needed, in order to properly understand the behavior of FFF products and thus to safely introduce them to the industry and engineering use. A good example of detailed fatigue life analysis is given in the literature by Poberezhnyi et al. [38], where authors, although on steel specimens taken from an offshore gas pipeline, point out that the complete picture of the fatigue failure of the material can only be obtained by the study of the macrocharacteristics of material and the microfractographic analysis of failed specimens, and then comparing those micro- and macro-scale data. The surface roughness of FFF polymer specimens is another parameter that needs additional attention when analyzing the fatigue life of FFF parts. The surface roughness of FFF samples can be reduced mechanically, by grinding or using special polishing equipment which uses alcohol liquids for removing layer lines. Thus, according to all aforementioned, the authors of the paper at hand will concentrate their future research on the fatigue performance of FFF-obtained polymer specimens in the following combinations:

- Comparison of different infill densities of FFF (3D printed) specimens and infill patterns and their influence on mechanical and fatigue properties;
- Different cycle rate values;
- Numerical modelling, using finite element method, based on proposed analytical models and findings;
- Fracture surface analysis;
- Surface roughness influence on fatigue fracture and cyclic durability;
- Influence of ultraviolet radiation and temperature on FFF polymer specimens.

Since the ASA is of weather-resistant polymer class, unlike ABS and PLA, the last stated fact is of particular importance in the exploitation of FFF and 3D printing of polymers in, for example, the shipbuilding and maritime industry, where the weather is very influential. It is, therefore, very interesting to study the impact of the mentioned factors on the fatigue performance of FFF-produced parts.

## Figures and Tables

**Table 1:** Physical properties of used polymer materials and filament [31,32,33].

Material/Property	PLA	ABS	ASA+
Density (g/cm^3^ at 21.5 °C; ISO 1183)	1.20	1.10–1.15	1.10
Melt Flow Rate (g/10 min)	210 °C/2.16 kg = 7 ÷ 11	220 °C/10 kg = 9 ÷ 14	260 °C/5 kg = 45
Melting temperature (°C)	150	-	228
Glass transition temperature (°C)	61	98.1	98

* PLA, polylactide; ABS, acrylonitrile butadiene styrene; ASA+, acrylonitrile styrene acrylate.

**Table 2:** Comparison of basic mechanical properties of PLA specimens.

Property	This paper	Manufacturer [31]	[Reference]
Young’s modulus (MPa)	2923	2623 ± 330	3340 [22]
Tensile strength (MPa)	32.1	46.6 ± 0.9	48.5 [22]

**Table 3:** Comparison of basic mechanical properties of ABS specimens.

Property	This paper	Manufacturer [32]	[Reference]
Young’s modulus (MPa)	2182	2174 ± 285	1960 ± 60 [5]; 1538 [21]
Tensile strength (MPa)	22.8	33.3 ± 0.8	32.8 ± 0.6 [5] 38.65 [21]

**Table 4:** Comparison of basic mechanical properties of ASA+ specimens.

Property	This paper	Manufacturer [33]	[Reference]
Young’s modulus (MPa)	1996	2020	1398.3 [34]
Tensile strength (MPa)	29.9	48	23 [34]

**Table 5:** Calculated values of *α* and *β* for the PLA material and given cycle rates.

Coefficient/Cycle Rate	10 Hz	20 Hz	30 Hz
*α*	1488.2	349.6	474.5
*β*	−0.4219	−0.227	−0.284

**Table 6:** Calculated values of *α* and *β* for the ABS material and the given cycle rates.

Coefficient/Cycle Rate	10 Hz	20 Hz	30 Hz
*α*	70,213.8	1133.9	236.8
*β*	−0.926	−0.487	−0.250

**Table 7:** Calculated values of *α* and *β* for the ASA+ material and given cycle rates.

Coefficient/Cycle Rate	10 Hz	20 Hz	30 Hz
*α*	3797.1	6574.1	753.6
*β*	−0.561	−0.671	−0.361

## Data Availability

The data included in this paper are available upon request to the corresponding author.
